# Efficient, high-throughput transfection of human embryonic stem cells

**DOI:** 10.1186/scrt23

**Published:** 2010-07-26

**Authors:** Jennifer C Moore, Kristin Atze, Percy L Yeung, Alana J Toro-Ramos, Cynthia Camarillo, Kevin Thompson, Christopher L Ricupero, Mark A Brenneman, Rick I Cohen, Ronald P Hart

**Affiliations:** 1 Stem Cell Research Center and Department of Cell Biology and Neuroscience, Rutgers, The State University of New Jersey, Room D251, 604 Allison Drive, Piscataway, NJ 08854, USA; 2 Lonza Cologne AG, Nattermannallee 1, 50829 Cologne, Germany; 3 Department of Genetics, Rutgers the State University of New Jersey, Room 325, 145 Bevier Rd, Piscataway, NJ 08854, USA

## Abstract

**Introduction:**

Genetic manipulation of human embryonic stem cells (hESC) has been limited by their general resistance to common methods used to introduce exogenous DNA or RNA. Efficient and high throughput transfection of nucleic acids into hESC would be a valuable experimental tool to manipulate these cells for research and clinical applications.

**Methods:**

We investigated the ability of two commercially available electroporation systems, the Nucleofection^® ^96-well Shuttle^® ^System from Lonza and the Neon™ Transfection System from Invitrogen to efficiently transfect hESC. Transfection efficiency was measured by flow cytometry for the expression of the green fluorescent protein and the viability of the transfected cells was determined by an ATP catalyzed luciferase reaction. The transfected cells were also analyzed by flow cytometry for common markers of pluripotency.

**Results:**

Both systems are capable of transfecting hESC at high efficiencies with little loss of cell viability. However, the reproducibility and the ease of scaling for high throughput applications led us to perform more comprehensive tests on the Nucleofection^® ^96-well Shuttle^® ^System. We demonstrate that this method yields a large fraction of transiently transfected cells with minimal loss of cell viability and pluripotency, producing protein expression from plasmid vectors in several different hESC lines. The method scales to a 96-well plate with similar transfection efficiencies at the start and end of the plate. We also investigated the efficiency with which stable transfectants can be generated and recovered under antibiotic selection. Finally, we found that this method is effective in the delivery of short synthetic RNA oligonucleotides (siRNA) into hESC for knockdown of translation activity via RNA interference.

**Conclusions:**

Our results indicate that these electroporation methods provide a reliable, efficient, and high-throughput approach to the genetic manipulation of hESC.

## Introduction

Human embryonic stem cells (hESC) are unique in their ability to continuously self-renew while maintaining the ability to differentiate into any cell in the adult body [1]. These characteristics give hESC the potential to be useful in many different aspects of basic and clinical research, including use as an *ex vivo *source for cellular transplantation; production of cells for screening drug candidates and assessing toxicity; and as an *in vitro *system for modeling human development and disease. However, the difficulty in producing genetically modified hESC lines has hindered the development of some of the most promising applications of hESC research (reviewed in [2]). Without the ability to easily and efficiently produce genetically modified cell lines, tools such as reporter cell lines, differentiation strategies based on the expression of certain growth factors, and the isolation of specific populations based on marker expression, will continue to limit the applications of hESC research.

The most common strategy for introducing exogenous nucleic acids into traditional, cultured cells is chemical transfection. In this process, DNA is introduced into the cells via liposomes or polycationic polymers (reviewed in [3]). While this process is relatively inexpensive and the vectors used have very few limitations, there are two major disadvantages. The first is that while many different chemical transfection strategies have been attempted to genetically modify hESC, the resulting efficiencies have generally been very poor, often significantly less than 1% (reviewed in [4]). An additional problem is that even when the exogenous DNA is successfully introduced into the cell, integration into the genome is poor, and when it does occur in hESC, the exogenous gene is often silenced, making isolation of stable transfectant cell lines inefficient [5,6]. The low efficiency of transfection combined with the inefficient integration of the construct into the genome makes the average efficiency for generating stable transfectant clones in hESC around 1 in 10^5 ^[5].

The bulk of successful genetic modification of hESC has been done with lentiviral vectors (reviewed in [4]). Lentiviral vectors are advantageous in several ways, most important is their ability to integrate into the cellular genome to produce stably expressing cell lines [7]. Lentiviral vectors have been shown to transduce many cell types (including primary and non-dividing cells) at high efficiencies and have been successfully used to transduce hESC [8]. Unfortunately, the use of these vectors has two major disadvantages. First, vector constructs larger than 11 kb package poorly, reducing the efficiency of transduction and effectively limiting the size of DNA that can be transferred [9]. The other disadvantage is the introduction of insertional mutagenesis. Since these vectors integrate randomly, they have the potential to activate oncogenes or inactivate tumor suppressor genes in the transduced cells, therefore raising safety concerns for potential clinical applications [10].

A third strategy for obtaining genetically modified cells is electroporation. In this method, DNA enters cells after an electrical current induces pore formation in the plasma membrane. While a disadvantage of this technique is reduced cell survival, electroporation has been successful in hESC [11-13]. As with chemical transfections, electroporation is not limited with respect to the size of DNA elements that can be inserted into the cells. Moreover electroporation is highly versatile in that it can be used to introduce other biological molecules such as exogenous proteins, mRNA or siRNA [14-18]. However, in most applications, electroporation has been difficult to scale for high-throughput usage.

Here we demonstrate that high throughput electroporation with either of two systems, the Nucleofection^® ^96-well Shuttle^® ^System (Lonza AG, Cologne, Germany) or the Neon System (Invitrogen, Carlsbad, CA, USA), can efficiently deliver exogenous DNA into H9 human ESC, and that the viability of the cells after transfection remains high. These systems were chosen because they might conceivably provide high-throughput applications--the Shuttle using a 96-well plate with well-by-well transfection and the Neon with convenient pipet tip-based transfection. We found that both instruments provide suitable transfections in hESC but that the Shuttle system had more consistent results for high throughput applications so we chose it for further investigation. To determine whether the methods developed will be generally suitable for hESC lines, we transfected several additional lines and found no significant differences in transfection efficiency or cell viability. We also demonstrate that siRNA oligonucleotides can be efficiently transfected to knock down protein expression.

As is the case with chemical transfections, DNA introduced by the Shuttle System is only rarely integrated into the genome. However, the high throughput capability of the Shuttle System allows the generation of large numbers of viable transfectants, increasing the chance that successful genomic integration can be recovered, as demonstrated by the generation of a stable cell line expressing red fluorescent protein (RFP). Although we made no attempt to directly compare promoters and antibiotic resistance markers, the process of generating stable lines allowed us to make some conclusions about the most effective promoter/resistance marker combinations. As reported by others, we also noticed that two of the most widely used viral drivers, CMV (cytomegalovirus immediate-early promoter) and SV40 (simian virus-40 early promoter/origin region) are less likely to confer stable expression in hESC [19,20].

Our study demonstrates that electroporation can be used in a high-throughput manner to produce either transient or stable transfectants in hESC with good viability. This will to be valuable for a variety of studies using hESC.

## Materials and methods

### Cell culture

Undifferentiated hESC (H9 [1], K306 [21], RNJ8 [22], and RNJ9 [22]) were cultured in the feeder-free system described by Ludwig and coworkers [23]. Briefly, cells were passaged every seven days at a splitting ratio of 1:6 to 1:10, so that the resulting cultures were 80 to 90% confluent on the day of passage. To passage, cells were loosened from the culture substrate with 1 U/mL Dispase (BD Biosciences, San Jose, CA, USA) and washed with DMEM/F12 medium, then gently scraped to completely remove the colonies from the substrate and break them into pieces. These pieces were transferred to new culture wells pre-coated with hESC-qualified Matrigel (BD Biosciences) and grown in mTeSR medium (StemCell Technologies, Vancouver, Canada). Starting 24 hours after passaging, the medium was replenished daily.

### Plasmid vectors

The vectors pEGFP-C, pRNAT-U6.1, and pTracer-BSD were purchased from Clontech (Mountain View, CA, USA), Genscript (Pistcataway, NJ, USA) and Invitrogen, respectively. pPGK-Puro was constructed by Peter Laird in the lab of R. Jaenisch [24]. pBM14 was constructed by Barbara Mitta in the lab of M. Fussenegger [25] and modified by insertion of a dsRed2 gene (details available upon request). pCMV-BSD was constructed by M. Brenneman (details available upon request).

### DNA electroporation with the Nucleofection^® ^96-well Shuttle^® ^System

Undifferentiated hESC were washed twice with PBS and detached from the substrate by five minutes of incubation with Accutase (StemCell Technologies) at 37°C. The detached cells were then dissociated by addition of 2 ml/well of mTeSR medium followed by gentle pipetting. The cells were pelleted by centrifugation at 800 × G for three minutes and resuspended to a density of 10 × 10^6 ^cells/ml in H9 Human ES Cell Nucleofector solution with the supplied supplement added (Lonza). For each electroporation, 400 ng of the plasmid vector pMaxGFP^® ^(Lonza) in a volume of 2 μl was placed in one well of a 96-well microcuvette plate (Lonza). A total of 20 μl of cell suspension (200,000 cells) was added to the well and pipetted to mix. Electroporation was done using program 96-CB-150 on the Shuttle System. After electroporation, the contents of each microcuvette well were dispersed as rapidly as possible with 80 μl of pre-equilibrated mTeSR media, then transferred to one well of a matrigel-coated 96-well plate. Transfections carried out with the Nucleofection shuttle system and compared to the Neon Transfection System were prepared as described above except that one million cells were used for each electroporation and the cells were plated on matrigel-coated 24-well plates after electroporation.

### Neon™ transfection

Undifferentiated hESC were prepared for transfection as described above but after dissociation cells were resuspended in 8 μl of Neon Resuspension Buffer R for every one million cells. For each electroporation, cells and 2 μl of pMaxGFP (200 ng/μl, Lonza) were aliquoted into a sterile microcentrifuge tube. A Neon Tip was inserted into the Neon Pipette and the cell-DNA mixture was aspirated into the tip avoiding air bubbles. The Neon Pipette was then inserted into the Neon Tube containing 3 ml of Neon Electrolytic Buffer E in the Neon Pipette Station. Cells were pulsed once with a voltage of 1,400 and a width of 20. After the pulse, cells were quickly transferred into a matrigel-coated culture plate containing pre-equilibrated mTeSR media.

### SiRNA electroporation

Transfections were done as described above with the exception that siRNA targeting green fluorescent protein (GFP) (Lonza) was added to the transfection mixture at levels of 200, 300 or 600 ng.

Twenty-four hours after transfection, the cells were washed twice with PBS and removed from the culture dish using Accutase (StemCell Technologies). Cells were then resuspended in 250 μl BSA/PBS/Azide buffer as described by Moore *et al. *[4]. Samples analyzed for SSEA4 expression were blocked for 15 minutes in DMEM/F12 (Invitrogen) containing 10% defined FBS (Hyclone, Logan, UT, USA) followed by incubation with phycoerythrin-labeled antibody to SSEA4 (R&D Systems, Minneapolis, MN, USA, catalog # FAB1435P) for 30 minutes. After washing with DMEM/F12, cells were resuspended in 250 μl BSA/PBS/Azide buffer. All samples were analyzed on a FACSCalibur flow cytometer (Becton Dickinson, San Jose, CA, USA), and the software program CellQuest, (Becton Dickinson, San Jose, CA, USA) was used for data acquisition and analysis. All values are reported as mean ± standard error of the mean.

### Viability

To determine the percentage of viable cells after transfection, the Vialight Kit from Lonza was used according to the manufacturer's protocol. This kit utilizes the ATP present in live cells in a luciferase-catalyzed reaction to produce light, which is measured and correlated to the number of live cells present. Briefly, 24 hours post-transfection, cells were lysed in 50 μl of cell lysis solution. After incubation for 15 minutes at room temperature, the cell lysates were transferred to a luminometer plate and mixed with 100 μl AMR PLUS reagent. After two minutes of incubation at room temperature in the dark, the plate was read on a luminometer. All values are reported as mean ± standard error of the mean for replicate samples.

### Immunofluorescence

Twenty-four hours post-transfection, cells in the 96-well plates were fixed with 4% paraformaldehyde at room temperature for 15 minutes, then permeabilized and blocked in 4% normal goat serum with 0.1% Triton X-100. Primary antibodies used were Oct4 (Millipore, Billerica, MA, USA, MAB4401 at 0.5 μg/ml) and SSEA 4 (Millipore, MAB4304 at 1.0 μg/ml). After washing with PBS, the cells were incubated with AlexaFluor conjugated secondary antibodies (Invitrogen/Molecular Probes) at 1:500 and visualized by fluorescence microscopy.

### Generation of stable transfectant lines

To generate stable transfectants, H9 or RNJ9 hESC cells were grown and handled as described above for transient transfection tests, but with the following modifications. After dissociation with Accutase, cells were resuspended to two-fold higher density, so that 400,000 cells were loaded into each well of the 96-well microcuvette together with 400 ng of linear or circular plasmid DNA. Immediately after electroporation, the cells from each well of the microcuvette plate were dispersed with 80 μl of mTeSR medium, then transferred to a single matrigel-coated well of a six-well culture tray containing pre-warmed mTeSR medium. Medium was replaced 48 hours after transfection and again at 72 hours. At 96 hours after transfection, medium was replaced and selective antibiotic (puromycin, blasticidin, geneticin/G418 or hygromycin) was added, at concentrations determined previously through kill curves. Medium and selective antibiotic were replenished daily at first, then less frequently as non-resistant cells were cleared from the culture. Individual colonies of resistant cells were allowed to reach 3 to 4 mm diameter, then transferred to smaller wells for initial expansion. Transfer was done by gently scraping a colony off the culture substrate, followed by incubation with a small volume of Accutase in a microfuge tube to dissociate the colony to a suspension of single cells, then replating to a single well of a 96- or 48-well tray prepared with matrigel and mTeSR medium.

### Statistics

Statistical significance was determined using a Student's *t*-test with paired equal variance and a two-tailed distribution. *P*-values less than 0.05 were considered significant.

## Results

### Efficient and high viability electroporation of H9 hESC

**Figure 1:**
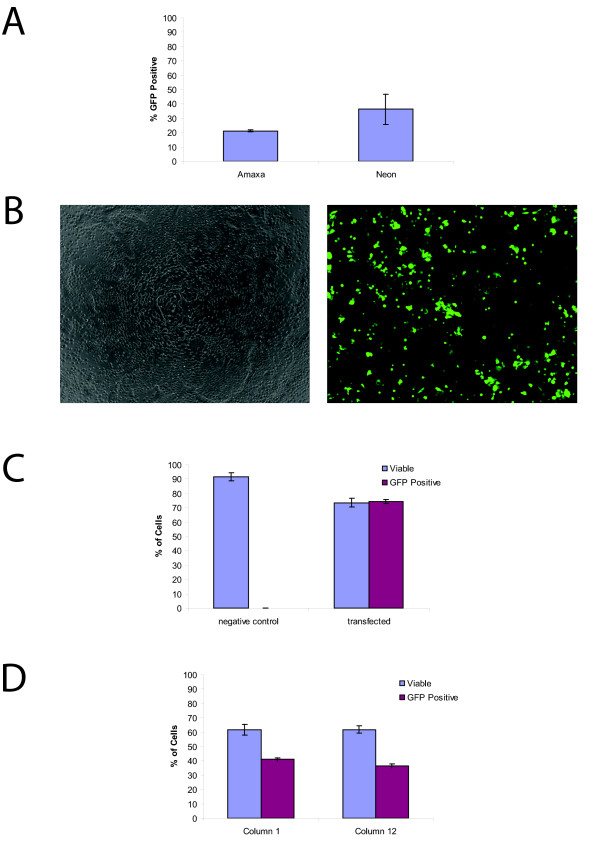
**Transfection of H9 is efficient and the resulting cells have a high viability**. **(a) **Comparison of the Neon and Shuttle^® ^Systems. Both systems can result in the efficient transfection of hESC. While the Neon system had a higher transfection efficiency, it also had increased variability between multiple transfections (36.2 ± 10.3% vs. 21.2 ± 0.8%). **b) **Transfected H9, phase contrast image (left panel) and GFP fluorescent image (right panel). **(c) **Quantification of Viability and Efficiency. FACS analysis demonstrates that after electroporation 73.5 ± 3.2% of the cells remain viable (compared to the 91.6 ± 2.7% viable in control reactions; *P *< 0.001) and that the transfection efficiency is 74.2 ± 1.4% (*P *< 0.001). **(d) **Viability and Transfection Efficiency in High Through-put Formats. All 96 wells of a shuttle plate were electroporated and the viability and efficiency of the first and last columns were compared. FACS analysis demonstrates that there is no change in viability between the first and last columns (61.8 ± 0.04% vs. 61.9 ± 0.03%, *P *> 0.05), however there is a small but significant decrease in transfection efficiency (41.0 ± 1.2% vs. 36.5 ± 1.3%, *P *< 0.05).

Two leading electroporation systems were tested for transfection efficiency and viability. In the initial tests, the Lonza Nucleofection^® ^96-well Shuttle^® ^System was compared with the Invitrogen Neon™ Transfection System since both systems required small volumes, few cells, and could conceivably be scaled for high-throughput screening. In both systems, a DNA vector (pMax-GFP) supplied with the Lonza Nucleofection kit and previously shown to be suitable for protocol optimization due to its high expression level and low toxicity was used. For the comparison of the two systems, transfections were done on a single population of cells. In each case, a single cell suspension of 1 million undifferentiated H9 hESC was mixed with 400 ng of the pMax-GFP DNA and electroporated. Although the Neon system had a higher mean transfection efficiency (36.2 ± 10.3% vs. 21.2 ± 0.8%), and the viability of the cells was similar 24 hours after transfection, variability between multiple samples was higher when using the Neon (Figure 1a). In order to determine any differences in the timing of transgene expression between the two systems, GFP expression was visualized by fluorescence microscopy every hour after transfection for six hours. Using both systems, GFP could be detected as little as one hour after transfection (not shown). While both systems were effective for hESC cultures, we chose to continue with the Shuttle System due to the more uniform transfection rates, similar viabilities, similar expression rates, but clearly simpler capability for performing high-throughput transfection when compared with the Neon system.

More through testing of the Shuttle System was performed to characterize its usefulness and reproducibility. This time 200,000 cells were mixed with 400 ng pMax-GFP and electroporated. GFP-expressing cells were visualized by fluorescence microscopy after six hours (Figure 1b). As a control, an equal number of cells mixed with vector DNA but not electroporated were also plated, and these had no visible GFP expression. At 24 hours post-transfection, pMax-GFP transfected (referred to as transfected cells) and cells mixed with DNA but not electroporated (control cells) were analyzed by FACS. GFP expression was detectable in 74.2 ± 1.4% of the transfected cells while the control cells had no significant GFP expression (*P *< 0.001) (Figure 1c). There is a large difference in transfection efficiency seen in Figure 1a and 1c but we believe this is due to the density of the cells before transfection. Our experiments suggest that there is an optimal density for transfection and this is further covered in the discussion. In order to determine the percentage of viable cells after transfection, the Vialight Plus Kit was used to measure ATP levels in both the transfected and control cells. The percentage of cells that remained viable after pMax-GFP transfection was 73.5 ± 3.2%, while the viability of the control cells was 91.6 ± 2.7% (Figure 1c). Although transfection significantly reduced cell viability (*P *< 0.001), the remaining viable cells are sufficient for most purposes and once transfected can easily be expanded (data not shown).

The ability to transfect hESC in a high throughput manner is an important first step in optimizing protocols for small molecule screens. In order to determine the suitability of the Shuttle System for this purpose, we compared the survival rate and transfection efficiency of cells electroporated in the first and last wells of a 96-well microcuvette shuttle plate. As seen in Figure 1d there was no statistically significant difference between the viability of cells electroporated in the first and last columns of a 96-well plate (61.8 ± 0.04% vs. 61.9 ± 0.03%, *P *> 0.05), however a small decrease in the efficiency of transfection was observed (41.0 ± 1.2% vs. 36.5 ± 1.3%, *P *< 0.05). Despite the small decrease in transfection efficiency, this demonstrates the utility of this technique for high throughput screens.

### Transfected H9 hESC retain expression of pluripotent markers

**Figure 2:**
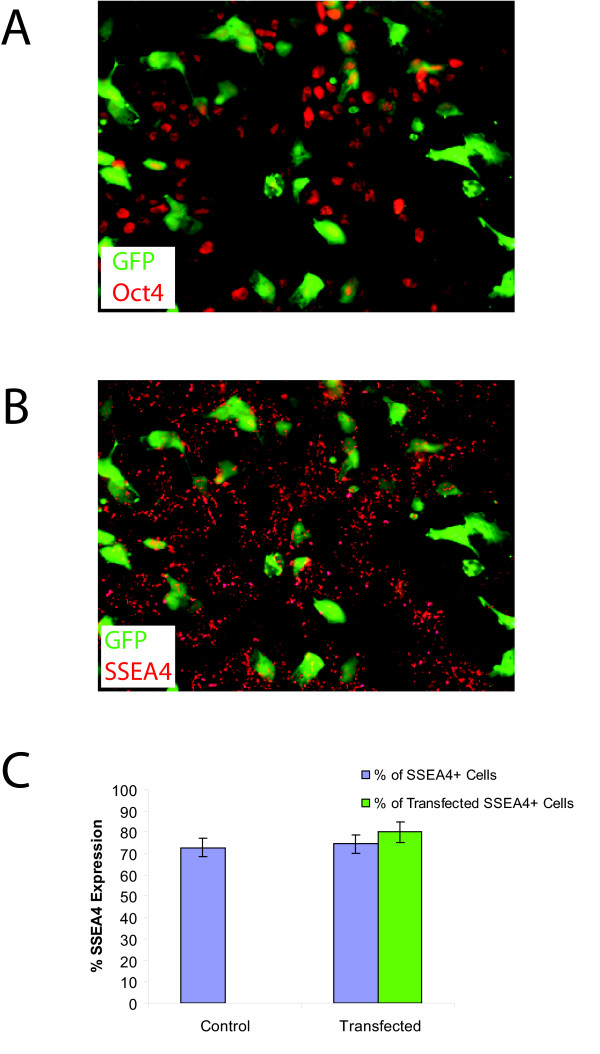
**Transfected H9 express markers of pluripotency**. **(a) **Fluorescence image of GFP transfected cells (green) that express Oct4 (red). **(b) **Fluorescence image of GFP transfected cells (green) that express SSEA4 (red). **(c) **FACS analysis for markers of pluripotency 24 hours after electroporation demonstrate that control and transfected cells expressed similar amounts of SSEA4 (blue bars; 74.4 ± 4.3% vs. 72.8 ± 4.4%; *P *> 0.5). In addition, of the GFP expressing cells 79.9 ± 4.7% also express SSEA4 (green bars).

The ability of hESC to maintain their pluripotency after transfection is required for developing successful protocols. To determine whether H9 cells continued to express pluripotent markers after transfection, the expression of Oct4 and SSEA4, two commonly used markers, was determined by immunofluorescence. As seen in Figure 2a, b, large numbers of electroporated cells (green in Figure 2) continued to be Oct4-positive (red in Figure 2a) and SSEA4-positive (red in Figure 2b) 24 hours after transfection, independent of their GFP expression. To quantitatively determine if the majority of cells retained pluripotency markers after electroporation, the percentage of SSEA4 positive cells were quantified by FACS. As seen in Figure 2c, 24 hours after electroporation, 74.4 ± 4.3% of electroporated cells expressed SSEA4, while 72.8 ± 4.4% of non-electroporated, control cells expressed SSEA4 (*P *> 0.5), demonstrating no difference in pluripotency between transfected and non-transfected cells. In addition, 79.9 ± 4.7% of cells that were expressing GFP 24 hours after transfection also expressed SSEA4^+^, which is similar to the rate found in the entire culture. The SSEA4-positive cells can potentially be separated from the differentiated cells by flow sorting or manual picking of colonies, allowing a pure culture of transfected, pluripotent cells to be propagated.

### Transfection of siRNA

**Figure 3:**
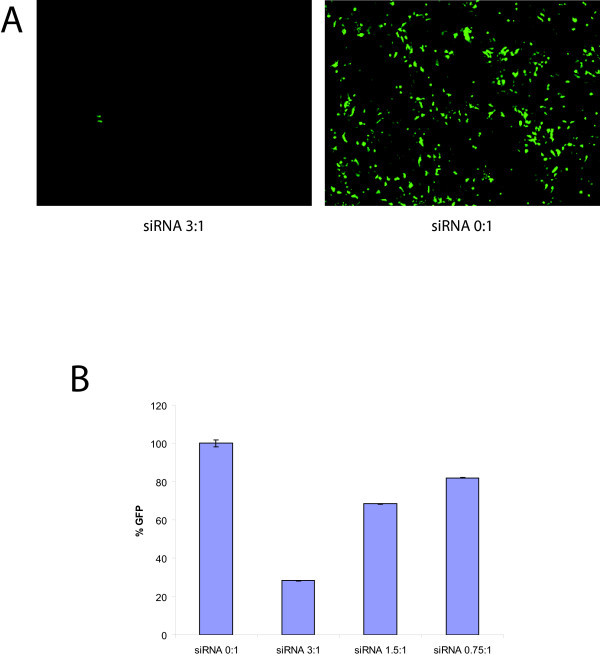
**Co-transfection with siRNA can suppress GFP expression**. **(a) **Co-transfection of a siRNA that targets GFP at a 3:1 mass ratio to pMax-GFP results in reduced GFP expression (left panel). Transfection with pMax-GFP only (right panel). **(b) **Quantification of GFP knock-down by FACS analysis demonstrates that siRNA to vector DNA ratios of 3:1,1.5:1 and 0.75:1 result in GFP reductions of 71.75%, 31.7% and 18.0% (*P *< 0.01, 0.01 and 0.05, respectively).

To determine whether the 96-well Shuttle^® ^System was also capable of efficiently delivering siRNAs for gene knock-down, the pMax-GFP plasmid was co-transfected with siRNA oligonucleotides that targeted the GFP transcript. The amount of siRNA needed to suppress GFP expression effectively was determined by titrating various amounts of siRNA with the pMax-GFP vector (siRNA to pMax-GFP mass ratios of 3:1, 1.5:1 and 0.75:1). Fluorescence microscopy showed that GFP expression was visibly reduced when a three-fold excess of GFP-targeting siRNA was co-transfected (Figure 3a). FACS analysis showed that an siRNA to DNA ratio of 3:1 reduced the percentage of cells expressing GFP by 71.75% when compared to cells transfected in the absence of siRNAs (*P *< 0.01) (Figure 3b). siRNA to DNA ratios of 1.5:1 and 0.75:1 also reduced the percentage of GFP-positive cells by 31.7 (*P *< 0.01) and 18.0% (*P *< 0.05), respectively (Figure 3b). This demonstrates that the system can also be used for the transfection of siRNAs to knockdown protein expression.

### Transfection efficiency in multiple hESC lines

**Figure 4:**
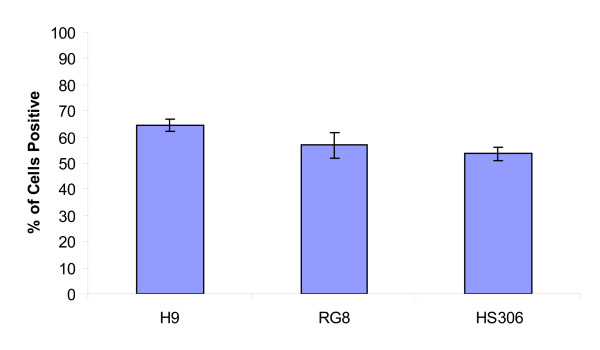
**Multiple hESC lines are transfected efficiently**. In addition to the H9 cell line, two hESC cell lines, RNJ8 and HS306, were also efficiently transfected, with resulting percentages of GFP-expressing cells greater than 50%.

To ensure that the transfection efficiency seen with H9 was not specific to that cell line, two additional hESC lines were tested. These lines were RNJ8 [22] and the HS306 hESC line [21]. Figure 4 shows the percentage of cells that were GFP positive by FACS analysis 24 hours after transfection (as described above). Transfection of the RNJ8 and HS306 lines resulted in 55.8 ± 4.8% and 53.6 ± 2.6% GFP-expressing cells, respectively, demonstrating that this method of transfection is suitable for hESC lines derived under different conditions by different laboratories.

### Generation of stable transfectant H9 cell lines

Isolation of stable transfectant clones requires some means to separate them from the larger fraction of cells that have not integrated vector DNA into their chromosomes. The simplest and most widely used method is antibiotic selection, which requires that a marker gene for antibiotic resistance be included in the vector DNA. Four different antibiotic resistance markers were tested in H9 cells: Puro (puromycin N-acetyltransferase), BSD (blasticidin-S deaminase), Hyg (hygromycin-B phosphotransferase) and Neo (neomycin phosphotransferase) (Table 1). These four markers are included in many commercially available mammalian expression vectors under the transcriptional control of various viral or mammalian promoter/enhancer sequences. To investigate the ability of the Shuttle System transfections to produce stable transformants, selectable markers driven by CMV or SV40 promoters were tested, in addition to markers driven by promoter/enhancer units from two mammalian genes; murine phosphoglycerate kinase (PGK) and human elongation factor 1-alpha (EF1α) (Table 1). Working doses for selection in H9 cells were established through a series of kill curves with puromycin, blasticidin-S, hygromycin-B and the neomycin analog geneticin (G418). The lowest dose that would completely kill an approximately 80% confluent well (24-well tray) of non-transfected H9 cells over five days or less was taken as a starting point for transfection trials.

**Table 1:** Efficiency of stable clone generation based on resistance markers and their promoters

Vector	Resistance marker	Transcriptional control	Selective drug (dose)	Frequency of stable integrants^5^
pPGK-Puro	Puro	PGK^1^	Puromycin (0.5 μg/ml)	>8 × 10^-5^
pTracer-BSD	BSD (GFP fusion)	EF1α^2^	Blasticidin-S (2.0 μg/ml)	4.9 × 10^-5^
pCMV-BSD	BSD	CMV^3^	Blasticidin-S (2.0 μg/ml)	6.3 × 10^-6^
pBM14	Neo	PGK^1^	Geneticin/G418 (50 μg/ml)	6.0 × 10^-6^
pEGFP-C3	Neo	SV40^4^	Geneticin/G418 (50 μg/ml)	6.3 × 10^-6^
pRNAT-U6.1 (GFP)	Hyg	SV40^4^	Hygromycin-B (10 to 100 μg/ml)	<1.0 × 10^-6 ^(none recovered)

^1^Murine phosphoglycerate kinase promoter/enhancer.

^2^Human elongation factor 1-alpha promoter/enhancer.

^3^Cytomegalovirus immediate-early promoter/enhancer.

^4^Simian virus-40 promoter/origin region.

^5^Values shown represent means of two to six experiments.

In order to maximize the recovery of stable transfectants, we found that the density of initial plating and the timing of selective drug application is crucial in hESC cells, since these cells apparently require close cell-to-cell contacts, and generally will not survive as a dispersed monolayer of separated cells. Our transient transfection experiments showed that GFP can be expressed in as little as one hour and this is presumably true for antibiotic resistance proteins as well. But if the selective drug is applied too early, stably transfected cells may become isolated and suffer from reduced survival rates as the non-resistant cells surrounding them die, despite their antibiotic resistance. To avoid this problem, we delayed the addition of selective antibiotics to the growth medium until 96 hours after transfection. In this way, resistant cells are afforded time to undergo multiple rounds of cell division, thereby forming a micro-colony of resistant cells before selection begins. Doing so required that the hESC cells be plated after transfection at a density high enough to allow the cells to reestablish cell-to-cell contacts and resume growth, but low enough to allow four days of growth without forming a confluent culture of fused colonies. To achieve this, the cells were generally re-plated after electroporation at a density of 400,000 cells/well in a six-well culture tray. Plating at densities of 300,000 cells/well or less resulted in very sharply reduced cell attachment and survival. Plating at densities of 600,000 cells/well or higher tended to result in cultures so dense after four days that it became difficult to clear the background of nonresistant cells and recover cleanly isolated colonies of resistant cells. Optimum plating densities for selection may prove to be somewhat different for hESC lines.

The frequency with which stable transfectant clones could be recovered varied among the selective drugs and vectors tested (Table 1). The best recoveries, 30 or more colonies per well, were achieved using puromycin selection, with a vector in which the Puro gene was driven by the mouse PGK promoter/enhancer. Blasticidin selection was also effective, with the best results from a vector with a BSD gene driven by the human EF1α promoter/enhancer. Although a vector with BSD driven by a CMV promoter/enhancer also yielded stable transfectants, the frequency was much lower. Geneticin (G418) selection allowed recovery of stable clones as well, with somewhat higher frequencies from a PGK-Neo vector than from an SV40-Neo vector. We were not successful in recovering stable transfectants by hygromycin selection, despite repeated attempts over a range of drug concentrations. Whether this reflects poor expression from the SV40-driven Hyg gene tested, low activity of the hygromycin-B phosphotransferase protein, or other factors in H9 cells is unclear.

**Figure 5:**
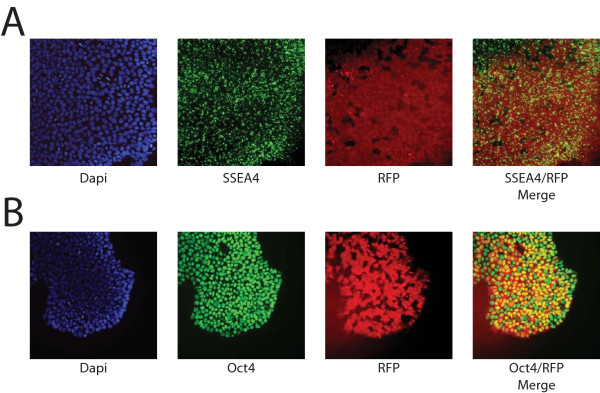
**Stable hESC cell line expressing red fluorescent protein**. RNJ9 cells transfected with EF1α-RFP/PGK-Neo were selected with 50 μg/ml G418 for two weeks after transfection and then grown for 10 passages. **(a) **Fluorescence images of dapi (blue), SSEA4 (green; in fixed cells SSEA4 staining appears punctuate due to the disruption of the cell membrane by paraformaldehyde, and RFP (red). **(b) **Fluorescence images of dapi (blue), Oct4 (green), and RFP (red).

In order to investigate the long term expression of multiple transgenes and their effect on pluripotency, RNJ9 stable transfectants expressing dsRed2 driven by the EF1a promoter and neomycin resistance driven by the PGK promoter were also isolated and maintained for more than 10 passages. During this time the cells maintained their RFP expression and immunofluorescence for Oct4 and SSEA4 confirmed their pluripotency (Figure 5a, b). This demonstrates that the Shuttle System is suitable for long term transgene expression, as well as bi-cistronic vector transfection, which is necessary for many applications such as reporter lines and lines harboring lineage specific genetic modifications.

## Discussion

The ability to genetically modify hESC with a high efficiency will provide new tools for understanding basic human development and tracking cells transplanted into animal models. In order to identify a method that can be used to efficiently electroporate hESC and be easily scaled for high-throughput applications, we tested the Nucleofection^® ^96-well Shuttle^® ^System from Lonza and the Neon™ Transfection system from Invitrogen. Both systems require low cell numbers, express transgenes in as little as one hour and are capable of efficiently transfecting hESC with a high viability. However, the higher reproducibility of the Shuttle^® ^System coupled with its ability to perform up to 96 individual transfections in batch mode led us to pursue the Shuttle over the Neon system.

Here we establish that the 96-well Shuttle^® ^System can be used to deliver DNA, as well as siRNA, and can generate genetically modified hESC lines. The greatest advantage of this system is the high transfection efficiency that can be achieved with a small number of cells in a high through-put manner. Although other groups have used electroporation to transfect hESC, the reported protocols required one to two million cells per transfection and were performed one transfection at a time [11,13,26,27]. Since the culture of hESC is time consuming and expensive it is advantageous that the number of cells necessary to achieve these high transfection efficiencies was only 200,000 per well and the viability of the transfected cells was often above 50%. These efficiencies were not cell line specific, as we were able to routinely transfect several independently derived hESC lines at efficiencies of at least 50% and sometimes as high as 70%. Interestingly, there seemed to be a correlation between initial density of the cells used for transfection and the transfection efficiency - the denser the cells were immediately before transfection, the less efficient the transfection. This may be due to the stress placed on cells that become too dense suggesting that highest transfection efficiencies are achieved when the cells are in the exponential growth phase. Since the Shuttle^® ^System uses a 96-well format, this method could be readily scaled up to perform multiple transfections in parallel for multiple replicates of multiple groups, or for screening large numbers of plasmids or siRNAs. To this end, we showed that the viability of the cells was not significantly decreased over an entire 96-well plate and that the transfection efficiency was only slightly reduced. Similarly, siRNA electroporation proved to be effective, at least on a gene carried by a co-transfected plasmid.

In order for genetically modified hESC to be usable in most applications, the modified cells must retain their pluripotency. We used immunofluorescence and FACS analysis to demonstrate the presence of pluripotency markers in the modified cells with, Oct4 and SSEA4. No statistically significant decreases in markers were observed between transfected and non-transfected cells, demonstrating that this method is suitable for expressing transgenes while likely maintaining pluripotency in both short term (transient transfection) and long term (stable transfection) experiments. It is important to note that more extensive characterization, such as karyotyping and teratoma formation of newly generated stable lines, would need to be done before the line could be formally considered to be pluripotent. Using this system we have also investigated the efficiency at which several different antibiotic selection cassettes and the promoters driving them can be used to derive stable cell lines. The majority of commonly used selection cassettes utilize either the CMV or SV40 promoters to drive their antibiotic resistance genes. However, several groups have reported difficulty in obtaining stable expression with these promoters, presumably due to gene silencing in both the undifferentiated hESC and hESC-derived differentiated cells [19,20]. Although we were able to use both the CMV and SV40 promoters to obtain stable lines, these occurred at a frequency which was at least 10-fold less than that of the EF1α and PGK promoters. Our data suggest that EF1α and PGK promoters are most efficient for the generation of stable lines and demonstrates that resulting lines can maintained in a pluripotent state for at least 10 passages.

## Conclusions

We have demonstrated that electroporation is a suitable method for reliable, efficient transfection of hESC and that the Lonza Nucleofection^® ^96-well Shuttle^® ^System can be scaled to high throughput methods while maintaining pluripotency. In addition to traditional DNA vector transfection, this method can also be used to transfect small RNA molecules to facilitate the knockdown of target genes. With these methods, the protocols necessary for a better understanding of stem cell biology can be generated, accelerating the application of clinical translation with these cells.

## Abbreviations

BSD: blasticidin-S deaminase; CMV: cytomegalovirus; EF1a: elongation factor 1-alpha; GFP: green fluorescent protein; hESC: human embryonic stem cells; Hyg: hygromycin-B phosphotransferase; Neo: neomycin phosphotransferase; PGK: phosphoglycerate kinase; Puro: puromycin N-acetyltransferase; siRNA: short interfering RNA; SV40: simian virus 40.

## Competing interests

Jennifer C Moore, Percy L Yeung, Alana Toro-Ramos, Cynthia Camarillo, Kevin Thompson, Christopher L Ricupero, Mark A Brenneman, Rick I Cohen and Ronald P Hart have no competing financial or personal interests in this work. Kristin Atze is an employee and shareholder of the Lonza Group, LTD, which holds patents on some of the technologies in this manuscript. Lonza did not contribute to the publication costs of this manuscript.

## Authors' contributions

Experiments were designed by JCM, KA, MB and RPH. Transfections were done by JCM, KA, PY, ATR, CC and KT. Immunofluorescence and microscopy were carried out by JCM and CLR. Human embryonic stem cells were provided by JCM and RC. The manuscript was written by JCM.
